# Prevalence of HPV infection among Thai schoolgirls in the north-eastern provinces in 2018: implications for HPV immunization policy

**DOI:** 10.1016/j.ijregi.2023.02.011

**Published:** 2023-03-04

**Authors:** Sompong Vongpunsawad, Chulwoo Rhee, Pornjarim Nilyanimit, Nimesh Poudyal, Suchada Jiamsiri, Hyeon Seon Ahn, Jinae Lee, Hyeong-Won Seo, Worrawan Klinsupa, Sunju Park, Nakorn Premsri, Chawetsan Namwat, Patummal Silaporn, Jean-Louis Excler, Deok-Ryun Kim, Lauri E. Markowitz, Elizabeth R. Unger, Supachai Rerks-Ngarm, Julia Lynch, Yong Poovorawan

**Affiliations:** 1 Centers of Excellence in Clinical Virology, Chulalongkorn University, Bangkok, Thailand; 2 International Vaccine Institute, Seoul, Republic of Korea; 3 Department of Disease Control, Ministry of Public Health, Nonthaburi, Thailand; 4 National Vaccine Institute, Bangkok, Thailand; 5 Centers for Disease Control and Prevention, Atlanta, Georgia, USA

**Keywords:** human papillomavirus, prevalence, genotype distribution, Thailand

## Abstract

**Objective:**

The aim of this study was to determine the prevalence of high-risk (HR) and vaccine-type human papillomavirus (HPV) infection among Thai schoolgirls who were not included in the national HPV immunization program.

**Methods:**

Cross-sectional surveys were conducted among grade 10 (15–16 years old) and grade 12 (17–18 years old) schoolgirls in two provinces of Thailand. Urine samples were collected using the Colli-Pee^Ⓡ^ device from November 2018 to February 2019. The samples were initially tested using Cobas^Ⓡ^ 4800. Subsequently, all Cobas-positive samples and 1:1 matched Cobas-negative samples were tested by Anyplex^Ⓡ^ assay. Prevalences of any HPV, any HR HPV, vaccine-type HPV, and individual HR HPV types were estimated by school grade.

**Results:**

Prevalences of any HPV and any HR HPV were 11.6% and 8.6% for grade 10, and 18.5% and 12.4% for grade 12 schoolgirls, respectively. Prevalences of bivalent vaccine-type HPV infection in grades 10 and 12 were 3.4% and 4.5%, respectively. Prevalences of quadrivalent and nonavalent vaccine-type HPV infections were 4.0%/6.6% and 6.4%/10.4% in grades 10 and 12, respectively. HPV16 was the most common type detected, followed by HPV58, 51, and 52. Circulating HR HPV types were similar between the school grades.

**Conclusion:**

A substantial burden of HR HPV infections was found among unvaccinated high school girls in Thailand.

## Highlights

• The prevalences of any high-risk HPV infection were 8.6% and 12.4% in grade 10 and grade 12 Thai girls, respectively.
• HPV16 was the most common type detected, followed by HPV58, 51, and 52.
• Prevalent high-risk HPV types were similar across school types and school grades.
• Multiple high-risk HPV infections were common, irrespective of school type.
• Catch-up HPV immunization for unvaccinated girls should be considered in Thailand.

## Introduction

Cervical cancer accounted for 342 000 deaths globally in 2020, with 90% of these occurring in low- and middle-income countries [1]. More than 95% of cervical cancer cases are attributable to human papillomavirus (HPV) infection [2]. Sexually active people can be infected with HPV throughout their lifetime; however, most HPV infections resolve spontaneously [3,4]. Nevertheless, every cervical HPV infection poses a risk of becoming persistent and, for high-risk types, oncogenic progression [5,6]. Initiation of routine cervical cancer screening and treatment programs has resulted in substantial progress in reducing cervical cancer incidence and mortality in high-income countries, while the introduction of HPV vaccination into the Expanded Program on Immunization (EPI) will provide powerful primary prevention [7]. However, these primary and secondary prevention measures have not been implemented in many low- and middle-income countries (LMICs), resulting in a disproportionate burden of cervical cancer [8].

The World Health Organization (WHO) adopted a global strategy towards eliminating cervical cancer in 2020, which calls for 90% of 9–14-year-old girls to be fully immunized with HPV vaccine by 2030. Currently, there are four WHO-prequalified highly efficacious HPV vaccines: two bivalent (HPV16, 18), one quadrivalent (HPV6, 11, 16, 18), and one nonavalent (HPV6, 11, 16, 18, 31, 33, 45, 52, 58). While HPV16 and 18 have been found to be the most detected genotypes globally, the prevalence of other high-risk (HR) HPV types is heterogeneous among continents and even among countries in the same region [9,10]. Therefore, HPV infection and HPV-related disease surveillance can be useful for tracking disease trends and assessing the impact of HPV vaccines [11]. Documenting the circulating HPV types in the population prior to and during vaccine implementation, especially HR and vaccine-preventable HPV types, is important because it can inform cervical cancer screening programs and national immunization policies, as well as guide development of future preventive and therapeutic interventions.

Thailand introduced HPV vaccine into the EPI in 2017, targeting grade 5 schoolgirls aged 9–10 years through a school-based program; however, multi-age cohort (MAC) catch-up for older girls was not included. Due to a global HPV vaccine shortage and the impact of the coronavirus 2019 (COVID-19) pandemic, Thailand's HPV immunization program was affected, resulting in no HPV immunization for 2019–2021 grade 5 cohorts. WHO's recent recommendations regarding the use of single-dose (SD) HPV for girls aged 20 years or younger, and the prioritization of MAC through 18 years of age [12], present financial and logistical advantages for MAC catch-up campaigns for girls who missed HPV vaccination at the routine age. Therefore, an analysis of data on the epidemiology of HPV among older Thai schoolgirls is warranted for exploring potential public health impacts. Currently, data on the distribution of HPV types among Thai schoolgirls in the age group prioritized for MAC by the WHO are limited. Therefore, our study aimed to describe HR HPV and vaccine-type HPV epidemiology among Thai high school girls, using a cross-sectional survey.

## Methods

The study was conducted as part of a larger community intervention study to measure the impact and effectiveness of SD and two-dose (2D) HPV vaccination in the north-eastern provinces of Thailand. The study methodology has been described in detail previously [13]. In brief, two regions in Thailand — Udon Thani and Buri Ram provinces — were chosen by the Ministry of Public Health (MoPH), and offered SD and 2D regimens, respectively. Grade 8 schoolgirls under 15 years old were offered the bivalent HPV vaccine in 2018. In parallel with vaccination campaigns, cross-sectional surveys were conducted among grade 10 (15–16 years old) and grade 12 (17–18 years old) schoolgirls in the same provinces to measure the baseline vaccine-type HPV prevalence. Baseline HPV prevalence was intended to serve as the reference population when vaccinated grade 8 schoolgirls entered grade 10 and grade 12 after 2 years and 4 years post-vaccination.

Since Thailand has two separate educational tracks for high school — general high school and vocational school — cross-sectional surveys were conducted in both school types. General high school grade 10 (G10) and vocational school year 1 (V1), and subsequently G12 and V3 are equivalent age cohorts. In each province, 2600 G10/V1 and 2000 G12/V3 schoolgirls were invited to join the survey from all participating schools. The target number of schoolgirls per school was calculated to be proportional to the school size in order to assure representativeness, and schoolgirls were systemically selected at the schools for enrollment.

After obtaining appropriate consent and assent, first-void urine samples were self-collected using a commercially available collection device (Colli-Pee^Ⓡ^; Novosanis, Wijnegem, Belgium), after providing brief training on the sample collection method. Urine samples were chosen for HPV genotyping to increase acceptability and feasibility of the study among schoolgirls. Each Colli-Pee contains 7 mL of urine conservation media as preservative and is designed to collect 20 mL of urine sample [14]. Collected urine samples were stored at 2–8°C and sent to the central laboratory for HPV testing, located at the Center of Excellence in Clinical Virology, Chulalongkorn University. All urine samples were processed within 96 hours of collection.

Upon receipt, 20 mL of urine sample was aliquoted into two tubes, containing 10 mL each. One aliquot was used for further processing while the other was stored at −20°C as a back-up. After centrifugation at 3000 rpm for 10 minutes, 9 mL of supernatant was discarded and the pellet was resuspended in the residual 1 mL of urine for HPV detection with a Cobas 4800^Ⓡ^ assay (Roche Molecular Diagnostics, Pleasanton, CA, v. 2.1.0), according to the manufacturer's protocol, which includes extraction and HPV detection. Cobas detects HPV16, HPV18, and aggregated results for 12 other HR HPV types (31, 33, 35, 39, 45, 51, 52, 56, 58, 59, 66, 68). Residual extracts from the Cobas 4800 in a 96-well format were retained for additional testing. Extracts from all Cobas-positive samples, and an equal number of negative samples (selected from the same plate and wells adjacent to a positive sample) were selected for testing with Anyplex^Ⓡ^ II 28 (Seegene, Seoul, South Korea) to individually identify 28 HPV types (HR HPV16, 18, 26, 31, 33, 35, 39, 45, 51, 52, 53, 56, 58, 59, 66, 68, 69, 73, 82, and low-risk (LR) HPV6, 11, 40, 42, 43, 44, 54, 61, 70), following the manufacturer's protocol.

For statistical analysis, the Cobas results for HPV16 and 18 were considered the outcome measure. Anyplex results were used for determining the other 12 HR HPV types for samples that were Cobas positive for the aggregated HR HPV types. Since only a subset of Cobas-negative samples was further tested using the Anyplex assay, HPV types not included in Cobas, i.e. potentially HR and LR HPV types, were extrapolated to the entire population, proportional to the available results. ‘Any HR HPV’ was defined as having one or more of 14 HR types included in the Cobas assay (HPV16, 18, 31, 33, 35, 39, 45, 51, 52, 56, 58, 59, 66, 68). ‘Any HPV’ was defined as having one or more of the 28 types detected by Anyplex.

Prevalences of HPV infections targeted by currently available WHO prequalified HPV vaccines were estimated based on these extrapolations. ‘HR HPV single infection’ was defined as a sample with only one HR HPV type detected, and ‘HR HPV multiple infection’ was defined as a sample with more than one HR HPV type detected. Total numbers of schoolgirls by school type at the time of survey were used to calculate weighted prevalences, using the Horvitz–Thompson adjustment method [15]. A confidence interval for each prevalence estimate was calculated using the Wilson method [16]. All statistical analyses were conducted using SAS software (version 9.4; SAS Institute, Cary, NC).

## Results

**Figure 1:**
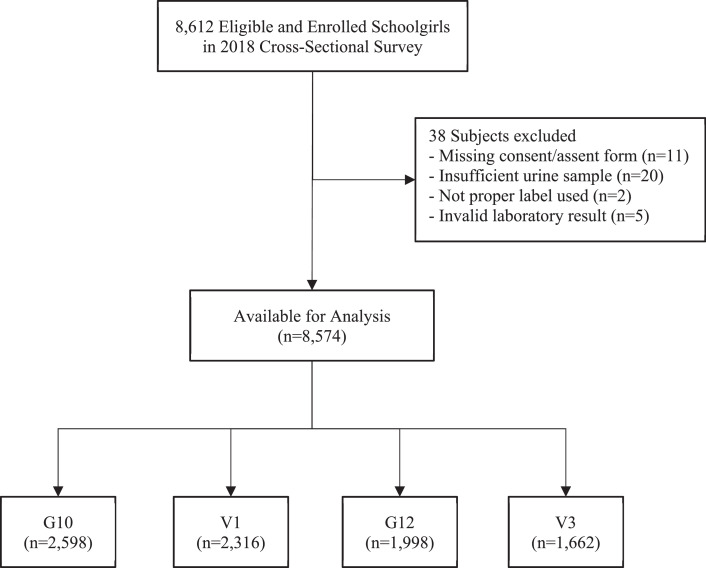
Consort diagram for subjects enrolled in 2018 cross-sectional survey.

In total, 8612 high school subjects were enrolled in the cross-sectional survey, which was conducted from November 2018 through February 2019. Subjects with protocol deviation or without laboratory results were excluded from the analysis, leaving 4914 schoolgirls for G10/V1 (2598 for G10 and 2316 for V1) and 3660 schoolgirls for G12/V3 (1998 for G12 and 1662 for V3). A consort diagram for subjects enrolled in the survey is shown in Figure 1.

G10/12: general high school grade 10/12; V1/3: vocational school year 1/3.

**Figure 2:**
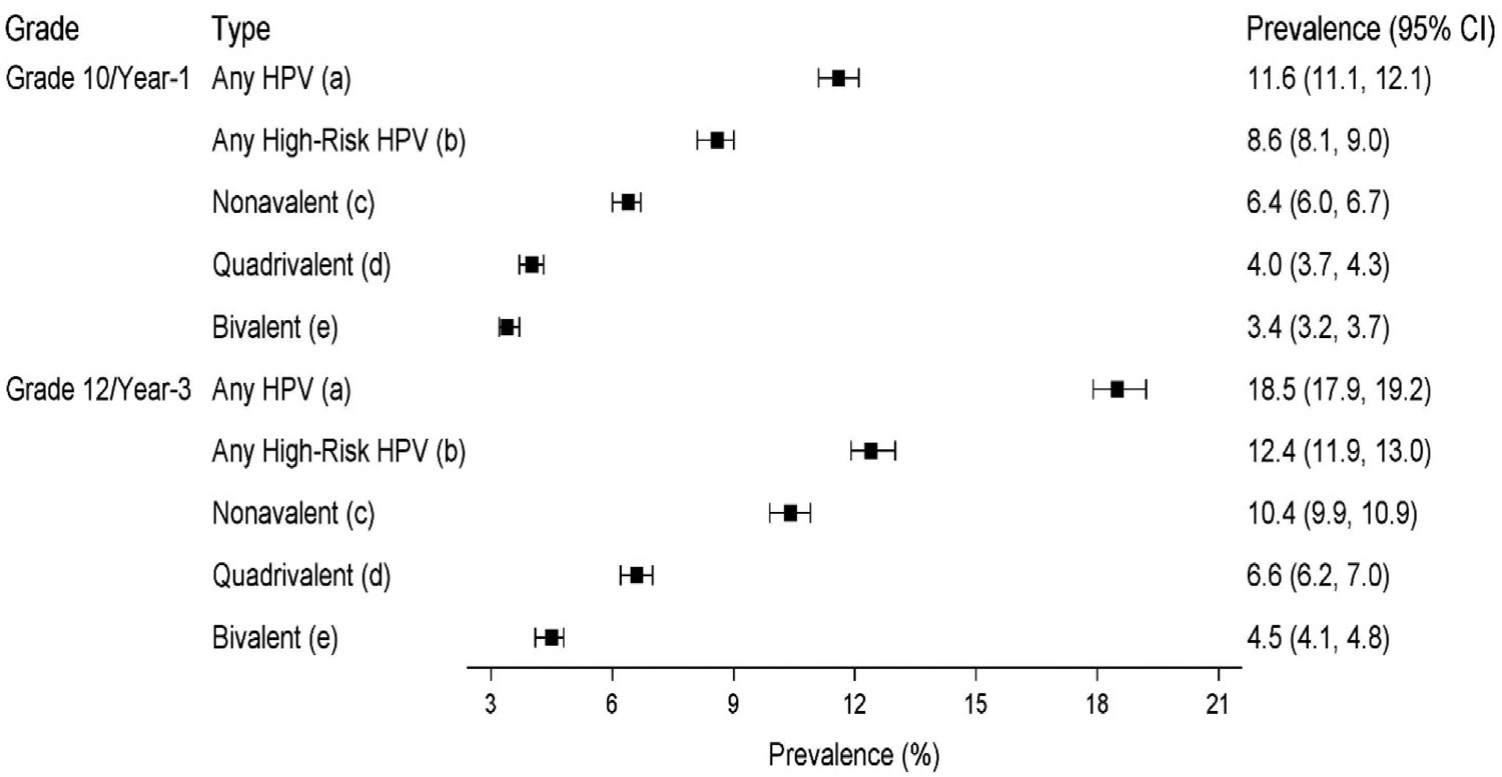
Weighted prevalences of any HPV, any high-risk HPV, and one or more vaccine-type HPV infections by school grade.

Note: Weighted prevalences were calculated by adjusting for sampling ratios in general high schools and vocational schools using Horvitz-Thompson adjustment. Confidence intervals (CI) were calculated using the Wilson score method. For HPV prevalences, including low-risk HPV types, Horvitz-Thompson adjustment was used for extrapolation to entire samples, based on available results. (a) Any HPV infection ..÷ positive for any one or more of the following HPV types: HPV16, 18, 26, 31, 33, 35, 39, 45, 51, 52, 53, 56, 58, 59, 66, 68, 69, 73, 82, 11, 40, 42, 43, 44, 54, 6, 61, 70. (b) Any high-risk HPV infection ..÷ positive for any one or more of the following HPV types: HPV16, 18, 31, 33, 35, 39, 45, 51, 52, 56, 58, 59, 66, 68. (c) Positive for any one or more of HPV16, 18, 6, 11, 31, 33, 45, 52, 58. (d) Positive for any one or more of HPV 16, 18, 6, 11. (e) Positive for any one or more of HPV 16, 18.

The weighted prevalence of any HR HPV infection was estimated at 8.6% (95% CI 8.1–9.0) in G10/V1 and 12.4% (95% CI 11.9–13.0) in G12/V3. A similar pattern of higher prevalence in G12/V3 was observed for any HPV infection (Figure 2). In G10/V1, the prevalences of bivalent vaccine-type, quadrivalent vaccine-type, and nonavalent vaccine-type HPV infections were 3.4%, 4.0%, and 6.4%, respectively. In G12/V3, the prevalences were 4.5%, 6.6%, and 10.4%.

**Figure 3:**
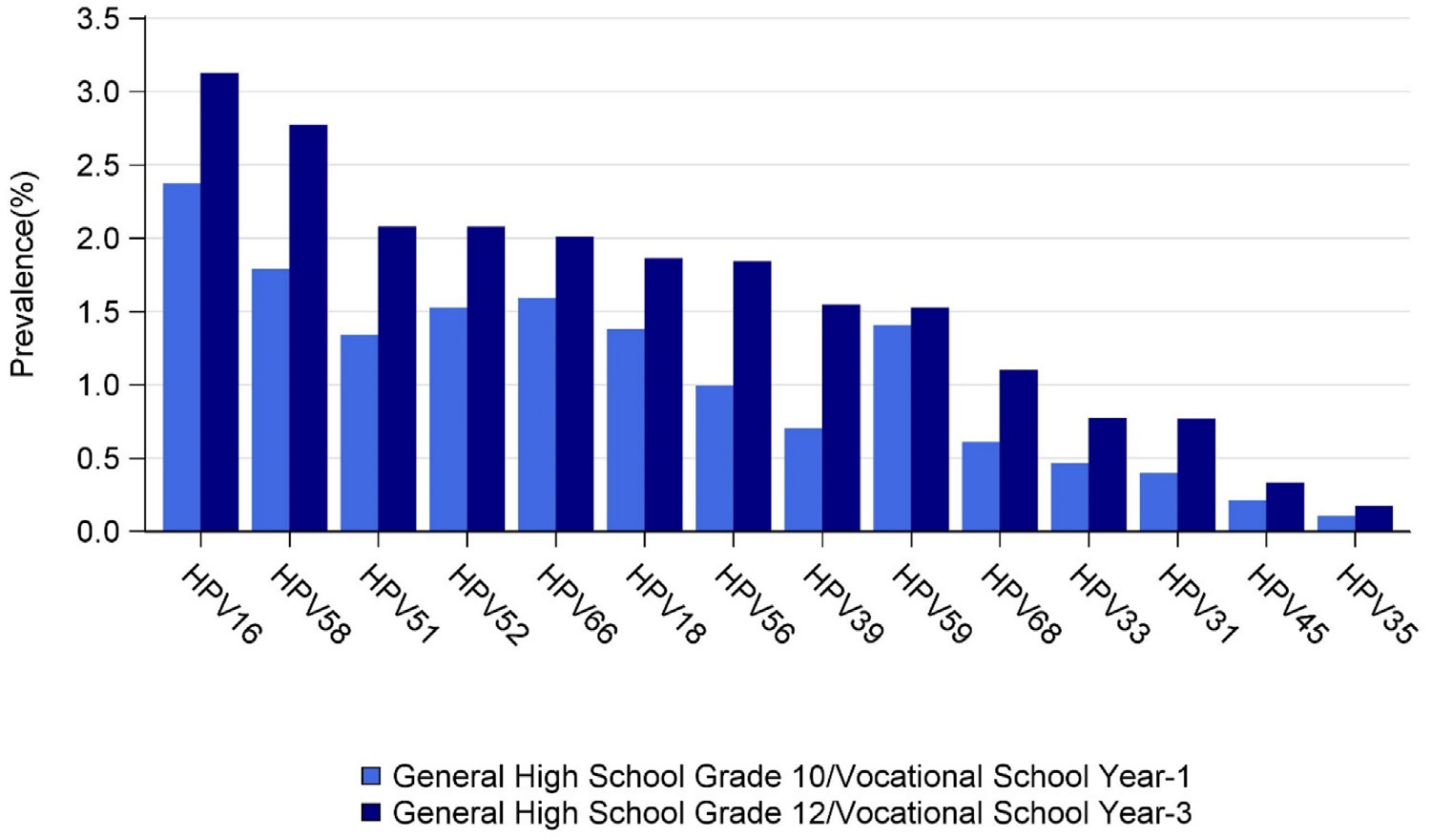
Weighted prevalences of high-risk HPV types, by school grade.

**Figure 4:**
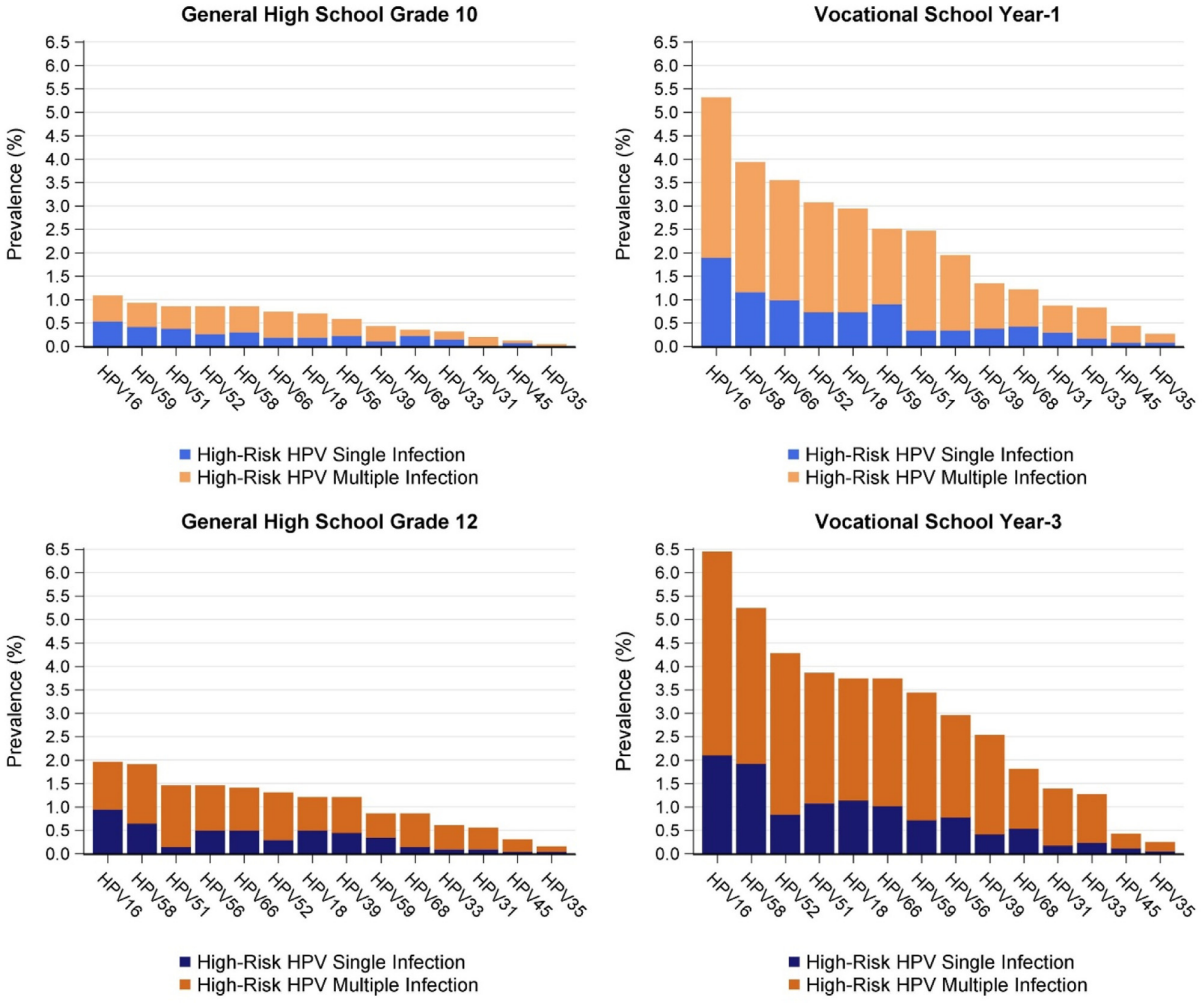
Prevalences of single and multiple high-risk HPV infections, by school type and grade.

Weighted prevalences for 14 individual HR HPV types are shown in Figure 3. Prevalences of HPV types were similar between the two school grades, but prevalences were higher in G12/V3 for all HR HPV types. The five most common HR HPV types were HPV16, 58, 51, 52, and 66. The prevalence of each HR HPV type, stratified by single and multiple infection, is shown by school type and grade (Figure 4). Although circulating HPV types were similar between school types, prevalences were generally higher in vocational schools than in general high schools for both school grades. Multiple HR HPV infections were common in both grades, irrespective of school type.

## Discussion

In this cross-sectional survey conducted in two north-eastern provinces of Thailand, a substantial burden of HPV infection was found among female high school students. Prevalences of any HPV infection were 11.6% and 18.5% for G10/V1 and G12/V3, respectively, indicating that by the time of high school graduation, almost one in five schoolgirls has at least one HPV infection. HPV infection was more common in vocational schools than general high schools, and prevalence was higher in older grades. However, circulating types of HR HPV were similar across school types and school grades.

This was the first study to report the distributions and prevalences of individual HPV types among schoolgirls in Thailand. HPV16 and 58 were found to be the most common HR HPV types, followed by HPV51, 52, 66, 18, and 56 for both school grades. Notably, for most HR HPV types, prevalences were two to three times higher in vocational schools than in general high schools. These data were consistent with the Ministry of Public Health 2019 sexual behavioral surveillance data on teenagers, which showed that the proportions of students reporting to have had sex were approximately 19% for general high school grade 11 and 41% for vocational school year 2 in 2019 [17].

Our findings indicated a higher HR HPV burden than previously reported estimates in Thailand. A study that enrolled randomly selected otherwise healthy women from two districts in Thailand from 1997 to 2000 reported a 5.6% prevalence for any HR HPV among women younger than 25 years of age [18]. Another study, which enrolled 5906 voluntarily registered apparently healthy women aged 20–70 years, either through hospital-based or population-based programs, from 2011–2012, reported a 15.1% overall HPV prevalence and a 6.4% HR HPV prevalence; HPV52 was the most frequent type, followed by HPV16, 51, and 58 [19]. In a 2014 study that assessed the distributions of HPV types in Thailand, HPV16, 18, 52, and 58 were reported to be the most common types; however, HR HPV prevalence was very low among women younger than 26 years of age [20]. Despite the differences in subject enrollment, methodology, and laboratory assays, similar HR HPV types — HPV16, 18, 52, and 58 — were consistently reported most frequently across multiple age groups in Thailand. Furthermore, HPV16, 18, 52, and 58 were reported to be the most common types among cervical cancer patients in Thailand [21].

HPV infection can vary depending on age, study setting, sexual behavior, and anatomical site. In addition, sample collection methods and assays can affect the types detected. While HPV detection is used as a surrogate for HPV infection, this is not completely accurate because detection at a single time point could indicate not only HPV infection but also recent deposition of HPV [22]. Since most HPV infections are expected to resolve spontaneously [4], HPV infection from a single time-point collection should be differentiated from persistent HPV infection, often defined as positive in two consecutive samples with an interval of at least 6 months. Persistent infection is a prerequisite for development of precancerous lesions. Nevertheless, single time-point HPV prevalence data are useful for assessing the impact of vaccine introductions and other public health programs.

Device-assisted self-collection of first-void urine was used to increase acceptability and feasibility of sampling for the study participants, as compared with traditional cervical or vaginal swab. When compared with cervical samples, pooled sensitivity and specificity of urinary detection of any HPV were estimated at 87% and 94%, respectively, in a previously conducted meta-analysis [23]. Although HPV detection from clinician-collected samples is known to be more sensitive than for self-collected urine samples [24], the latter can serve as an acceptable alternative sample type for population-based cross-sectional surveys, especially among young girls. Colli-Pee urine sample collection has been used in previous studies that aimed to measure HPV prevalence [25,26]. Prior to the study implementation, a pilot study comparing HPV results in urine samples versus clinician-collected swabs found 97% concordance between urine and swab (data not shown).

When Thailand introduced HPV vaccine into the EPI in 2017, G10/V1 and G12/V3 schoolgirls enrolled in this study were not eligible for the national program. Because the number of young women who self-purchase HPV vaccine is extremely low (< 2%) in Thailand [27], the HPV prevalence found in our study represented the prevalence prior to HPV vaccine introduction. Although HPV prevalence was not measured among lower grades in our study, considering the prevalence of HR HPV infection among G10/V1 girls, and the median age of sexual initiation in Thailand [28,29], HPV vaccination of grade 5 girls aged 9–10 years would prevent most infections and offer the greatest benefit. However, based on our data, a MAC campaign in older schoolgirls would still offer substantial public health benefits, and the recent simplified HPV vaccination recommendation would make this more feasible.

The prevalence of nonavalent vaccine-type HPV infection was approximately two times higher than that of the bivalent vaccine-type for both school grades. A study that assessed the impact of quadrivalent HPV vaccine use in Thailand showed it to be cost-effective with or without catch-up immunization [30]. Our findings demonstrated a substantial burden of HR HPV infection that is not targeted by bivalent or quadrivalent HPV vaccines. Since the prevalence of HPV infection does not directly reflect the proportions of disease caused by different HPV types, HPV-related disease burden studies will be important. Further studies on the cost effectiveness of using nonavalent HPV vaccine in the immunization program are warranted.

Our study was subject to several limitations. First, although it involved a high representation of schoolgirls from two north-eastern provinces, our finding cannot be generalized to other regions of Thailand, especially to the capital city Bangkok, where schoolgirls may have different sexual behaviors. Second, schoolgirls were enrolled irrespective of prior HPV immunization history, and therefore the participants may have included some who had self-purchased HPV vaccine. However, the proportion of schoolgirls who self-purchased HPV vaccine was expected to be low [27], and therefore have a minimal impact on HPV prevalence. Lastly, our study sample size was not sufficiently powered to provide estimates for individual uncommon HR HPV type infections.

## Conclusion

This study reported prevalences of HR HPV infections among unvaccinated high school girls in Thailand. MAC catch-up HPV immunization for older schoolgirls could provide protection for girls who were not eligible for the routine vaccination program.

## Ethical approval statement

The authors declare that they conducted this study ethically and in accordance with the World Medical Association Declaration of Helsinki. All study participants were informed about the study and voluntarily signed the written assent/consent form. The study protocol was approved by the Thailand Ministry of Public Health Ethical Committee (EC), the International Vaccine Institute (IVI) Institutional Review Board (IRB), and the Chulalongkorn University EC. The US Centers for Disease Control and Prevention (CDC) IRB deferred to the IVI IRB, based on a reliance agreement.

## Conflicts of interest

The authors declare that they have no competing interests.

## Data Availability

Deidentified individual participant data that underlie the results reported in this article including data dictionary and analytic code will be publicly available. Data will be found at https://figshare.com.
